# Intra-Tissue Pressure Measurement in *Ex Vivo* Liver Undergoing Laser Ablation with Fiber-Optic Fabry-Perot Probe

**DOI:** 10.3390/s16040544

**Published:** 2016-04-15

**Authors:** Daniele Tosi, Paola Saccomandi, Emiliano Schena, Dinesh Babu Duraibabu, Sven Poeggel, Gabriel Leen, Elfed Lewis

**Affiliations:** 1 School of Engineering, Nazarbayev University, 53 Kabanbay Batyr, Astana 01000, Kazakhstan; daniele.tosi@nu.edu.kz; 2 Institute of Image-Guided Surgery (IHU), S/c Ircad, STRASBOURG Cedex, Strasbourg 67091, France; 3 Unit of Measurements and Biomedical Instrumentation, Universita’ Campus Bio-Medico di Roma, via Alvaro del Portillo 21, Roma 00128, Italy; e.schena@unicampus.it; 4 Optical Fibre Sensors Research Centre (OFSRC), University of Limerick, Limerick V94 T9PX, Ireland; dineshbabu.duraibabu@ul.ie (D.B.D.); sven.poeggel@ul.ie (S.P.); gabriel.leen@ul.ie (G.L.); elfed.lewis@ul.ie (E.L.)

**Keywords:** fiber optic sensors, pressure measurement, Fabry-Perot, ablation of tissue

## Abstract

We report the first-ever intra-tissue pressure measurement performed during 1064 nm laser ablation (LA) of an *ex vivo* porcine liver. Pressure detection has been performed with a biocompatible, all-glass, temperature-insensitive Extrinsic Fabry-Perot Interferometry (EFPI) miniature probe; the proposed methodology mimics in-vivo treatment. Four experiments have been performed, positioning the probe at different positions from the laser applicator tip (from 0.5 mm to 5 mm). Pressure levels increase during ablation time, and decrease with distance from applicator tip: the recorded peak parenchymal pressure levels range from 1.9 kPa to 71.6 kPa. Different pressure evolutions have been recorded, as pressure rises earlier in proximity of the tip. The present study is the first investigation of parenchymal pressure detection in liver undergoing LA: the successful detection of intra-tissue pressure may be a key asset for improving LA, as pressure levels have been correlated to scattered recurrences of tumors by different studies.

## 1. Introduction

Thermal ablation (TA) has gained significant interest from clinicians and scientific community, for the mini-invasive treatment of tumors [1,2,3,4,5,6]. The principle of operation of TA relies on a miniature, typically percutaneous, applicator on active tip(s) positioned at the point of treatment, and connected to an electromagnetic source: a spatially confined heat field is induced in proximity to the active tip(s), and propagated to the adjacent tissue [1]. Methods using radiofrequency ablation (RFA) [2,3], microwave ablation (MWA) [4], high-intensity focused ultrasound (HIFU) [5], and emerging techniques based on gold nanoparticles and light-activated nanoparticles [6] have been reported for ablation of tumors. With respect to resection, TA methods provide minimal invasiveness for patients, often resulting in outpatient care, and enable repeatable treatments.

Laser ablation (LA) stands as the main alternative to electrical-based treatments [7,8,9,10]. LA makes use of a mid-power laser source, coupled in an optical fiber that serves as applicator, which is positioned inside the tumor. Applications of LA to hepatic [7], pancreatic [8], thyroid [9], and brain tumors [10] have been reported. Compared to mini-invasive RFA [2], LA yields an even smaller invasiveness, due to the microscopic size of the applicator, and higher resilience to cellular vaporization [7,8]. Moreover LA can be performed under echo ultrasound guidance, without percutaneous access [11]

It is expected that LA will sustain a substantial effort to improve performances within the next years, thanks to the development of fiber lasers, dynamic beam control, and micro-structured fiber applicators [12], which may allow unprecedented control of the ablation pattern at the micro-scale. In addition, the cost-effective outline of LA is having significant impact on healthcare in emerging countries, as this procedure has an estimated compound annual growth rate around 18% in Asia-Pacific and Eurasia [13].

Despite the premises, there are some limitations that still prevent TA, and particularly LA, to outperform surgical resection in cancer care [7,8]. A major restriction is the lack of biophysical control during the ablation process, in order to have a real-time benchmark and therefore being able to dynamically adjust ablation parameters [8,14]. To overcome this barrier, a recent research trend is the application of optical fiber sensors (OFS) for thermal monitoring in mini-invasive RFA [15] and LA [14]; to date, OFS-based techniques for spatially resolved temperature detection have been pioneered [14,15].

The measurement of intra-tissue pressure, however, is an open task. Pressure is a key parameter for the treatment of encapsulated tumors, such as hepatocellular carcinoma (HCC) [16,17,18]. The rise of pressure during RFA has been correlated by different studies [16,19] with increased risk of intrahepatic and extrahepatic seeding and dissemination of tumor: Inokuchi *et al*. [19] report that excessive increase of pressure during the early stage of ablation may induce tumor cells to dislocate. Among others, the study of Mulier *et al*. [18] shows that high intra-tumoral pressure causes scattered recurrences [20], which reduce long-term survival rates. However, some procedural solutions, mostly based on the multi-step control of the delivered energy, demonstrated to prevent increased intrahepatic pressure during RFA [16,17] and, recently, during MWA [21].

Technological limitations have prevented successful intra-tumoral pressure detection. This measurement is extremely demanding, from metrology point of view: (1) the pressure sensor must have a microscopic footprint and cabling size, and it must not alter the ablation pattern; (2) compliance to the ISO10993 biocompatibility standard is required for operating *in vivo*; (3) the sensor must have sufficient mechanical strength to support insertion and removal from the tissue; (4) the sensor must be thermally insensitive, as it operates in a region where both pressure and temperature vary abruptly, spatially and temporally [1,16]. The latter requirement is the most demanding, as both micro-electromechanical systems (MEMS) and OFS probes are often cross-sensitive to both pressure and temperature [22]; even OFS probes that mount a temperature compensator [23] are not effective as, due to the high thermal gradients, the two sensors would operate at different temperatures, making compensation impractical.

The first measurement of pressure in RFA was performed in 2005 by Kotoh *et al.* [16], using a MEMS sensor. The measurement was performed in a phantom confined in a sealed box, while pressure recording was performed outside the box, at 3 cm distance from the ablation peak. The MEMS probe did not satisfy all requirements, as it was not minimally invasive, nor thermally insensitive: therefore measurements were performed far from the point of treatment. Nevertheless, a protocol for optimal RFA was developed [17] and adopted in clinical environments, and different RFA devices were compared in terms of intra-tumoral pressure [24]. In 2014, Tosi *et al*. proposed a methodology for pressure detection based on a fiber-optic Extrinsic Fabry-Perot Interferometer (EFPI) method in RFA [25]. This approach, for the first time, satisfies all the four requirements for successful pressure detection, and the experimental results are in disagreement with [16,17], in terms of maximum pressure detection and pressure spatial gradients. So far, all pressure measurements have been limited to RFA and MWA; to the best of our knowledge, no measurement of intra-tissue pressure have been reported for LA.

In this investigation, we report for the first time a parenchymal pressure measurement performed during LA of a hepatic tissue, with a fiber-optic EFPI probe. The thermal insensitiveness of the probe allows us recording pressure at the point of treatment, providing a substantial improvement over far-field measurement as in [16,17]. Measurements have been performed on *ex vivo* on porcine liver, with a setup that mimics *in-vivo* operation. The obtained results are compared with the previous literature, and discussed in view of setting the basis for real-time intra-tumoral pressure measurement in clinical operation.

## 2. Experimental Setup

**Figure 1:**
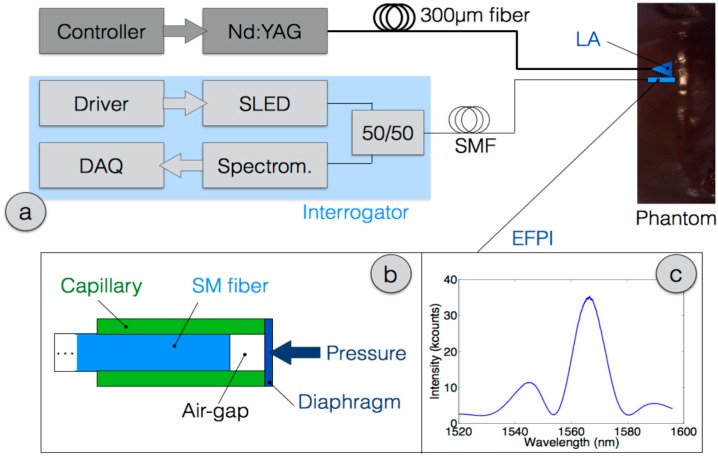
Schematic of the LA on animal phantom, and EFPI pressure detection: (**a**) Schematic of the system; (**b**) Sketch of the EFPI all-glass probe; (**c**) Spectrum of the EFPI probe.

The experimental setup for LA and pressure detection is shown in Figure 1, whereas LA and pressure measurement operate as separate blocks. LA is performed with a solid-state Nd:YAG laser (Echolaser X4, Elesta s.r.l., Florence, Italy), activated through a laser controller that allows selecting the output power. The laser emits around 1064 nm, and is operated in continuous wave. The applicator is a large-core fiber, having 300 μm diameter. Ablations were performed on freshly excited hepatic porcine tissue, which were obtained from locally bred pigs and stored in a refrigerator prior to perform LA experiments.

The EFPI probe, depicted in Figure 1b, has been fabricated as an all-glass, biocompatible assembly. The structure has been fabricated by means of fiber splicing and polishing, both manually performed. In order to preserve the all-glass structure, the pressure-sensitive diaphragm is a glass optical fiber (multi-mode fiber 62.5/200 μm); the Fabry-Perot cavity is formed within the standard single-mode input fiber and the multi-mode fiber serving as diaphragm. This arrangement allows a good fabrication control, as the diaphragm and capillary have same outer thickness and a very similar composition, which allows to be treated in a glass splicing machine. Initially, the multi-mode fiber is spliced to a quartz capillary, having 130 μm inner diameter and 200 μm outer diameter. The capillary was chosen as in [25]. With a manual splicer (Siecor X77), a single mode fiber SMF-28 (10/125 μm) is pushed within the hollow capillary, leaving a gap of about 38 μm length, that will serve as Fabry-Perot cavity, obtained between the multi-mode and single-mode fibers. All fibers are λ/10-polished prior to being spliced. The obtained cavity structure is subsequently adjusted to pressure sensing by reducing the thickness of the multimode fiber: in first step, the diaphragm is shortened to ~10 μm by means of fiber polishing, and subsequently wet-etching is performed shrinking the diaphragm thickness to approximately 2 μm. The resulting spectrum is shown in Figure 1c, as detected by the spectrometer.

The EFPI interrogator has been assembled in house as a portable device. A superluminescent LED (SLED, Exalos 2100 series) is used as light source, having 1 mW power on 60 nm bandwidth, and a Gaussian spectral profile; the SLED is powered by its driver board (Exalos EBD5200). Light from the optical source is directed to the probe through a 50/50 fiber-optic coupler. Light reflected by the EFPI is collected with a high-speed spectrometer (Ibsen Photonics I-MON-512-USB), operated on the 1520–1596 nm bandwidth. A LabVIEW™ program has been developed for data acquisition (DAQ), recording data from the spectrometer and switch and displaying the estimated output pressure; the software adjusts also the integration time of the spectrometer. The software also compensates the uneven spectrum of the SLED source, and adjusts the sampling frequency to approximately 10 Hz.

**Figure 2:**
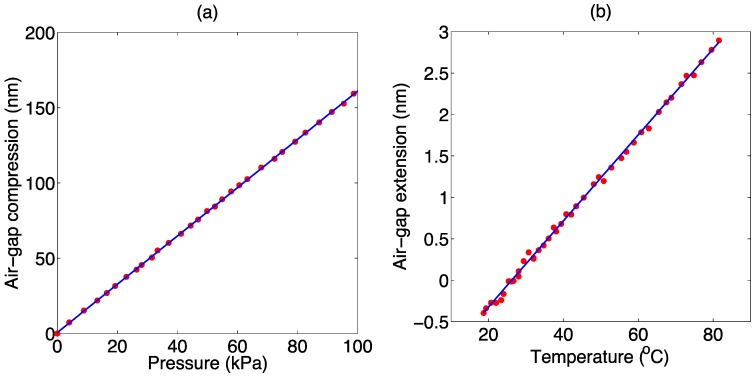
EFPI probe calibration. (**a**) Pressure calibration, reporting the Fabry-Perot cavity compression as a function of pressure, applied in an air tank at constant temperature; (**b**) Temperature calibration, reporting the cavity expansion factor as a function of applied temperature, in a thermal chamber, with no pressure variation.

EFPI calibration was performed in an air-filled tank, testing for pressure levels up to 100 kPa, and dynamically using a 60-cm burette filled with water, for fine detection of pressure up to 5.8 kPa. The pressure sensitivity, measured as air-gap compression as a function of applied pressure, is estimated as 1.61 nm/kPa, very close to [23] (1.5 nm/kPa) and [25] (1.6 nm/kPa). Figure 2a reports the pressure calibration performed in the air-filled tank, with pressure ranging from 0 kPa to 100 kPa, measured with a reference MEMS sensor (reference 0.1 kPa). Thermal effects were recorded by placing the probe in a temperature chamber, in absence of pressure changes; a low thermal sensitivity has been recorded, estimated as −0.05 nm/°C. Thus, a thermal detuning of −0.03 kPa/°C was obtained, which is to the best of our knowledge one of the lowest values for miniature-sized fiber optic pressure probes in absence of temperature compensation (approximately 350 times smaller than [23]). Figure 2b reports the thermal calibration performed in a temperature chamber, from 18 °C to 82 °C; the slope of the curve is estimated as 52.2 pm/°C. In absence of fluctuations of refractive index of the outer medium, pressure measurement yields accuracy better than 0.1 kPa [25]. The results of the calibration are in line with the previous pressure sensing system presented in [25] and developed by University of Limerick, which makes use of the same splicing method and capillary/diaphragm structure.

**Figure 3:**
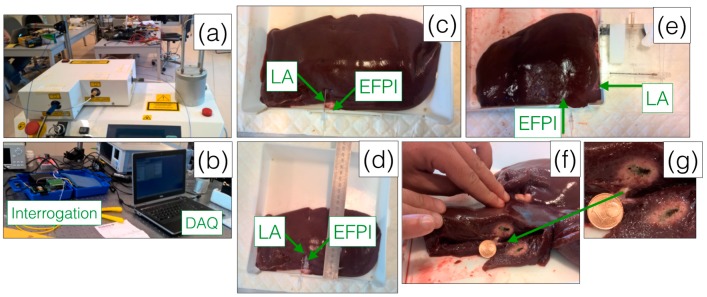
Picture of experimental setup and LA outcomes. (**a**) Medical-grade Nd:YAG laser, with 4 outputs; (**b**) EFPI interrogation box, and data acquisition (DAQ) via software; (**c**–**e**) Positioning of EFPI and LA inside the animal phantom, after insertion needle is removed (**c**), and prior to needle removal (**d**) with parallel positioning, and for perpendicular positioning (**e**); (**f**) Outcome of LA experiment, referenced to a 1€ cent coin (16.25 mm diameter); (**g**) Zoom of ablated tissue.

Figure 3 shows a series of photographs of the experimental setup, fiber insertion, and ablation outcome. In particular, the insertion of the EFPI probe into the tissue is shown: a needle is firstly inserted to guide the fiber into the tissue, and subsequently removed to leave the fiber in place. With this setup, no fracture or damage to the probe, despite its miniature size, was observed. The setup mimics *in-vivo* insertion of a fiber-optic probe.

## 3. Experimental Results

A set of four different experiments of LA, with simultaneous pressure monitoring, has been performed. Table 1 summarizes the parameters of the setup used in each experiment. Different levels of Nd:YAG laser optical power have been set, ranging from 3 W to 5 W; in each experiment, the laser was turned on for different durations, ranging from 52 s to 105 s. The tip-to-tip distance is defined as the geometrical distance between the LA applicator (center of the optical fiber connected to the Nd:YAG source) and the EFPI sensor (center of the tip-mounted diaphragm); thus, by increasing the tip-to-tip distance, the measurement is performed further away from the ablation point [8]. As the LA applicator and the pressure probe were positioned in a deep-seated position inside the tissue, the estimation of the tip-to-tip distance was made by measuring the geometrical distance between the probes from the pictures taken before and after each experiment. We positioned the fibers such that the tip-to-tip distance ranges from 0.5 mm to 5 mm, in order to verify pressure values and its temporal evolution in different parts of the tissue, with respect to the LA applicator tip. Three experiments were performed positioning the EFPI probe on the side of the LA fiber applicator, with the two fibers placed in parallel, as in Figure 3c,d, while a fourth experiment was carried out positioning the EFPI along the perpendicular direction to the applicator, as in Figure 3e.

**Figure 4:**
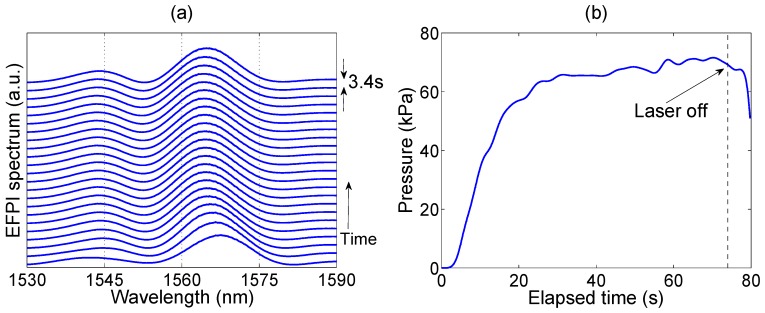
First experiment in pressure detection in LA. (**a**) The chart reports EFPI spectrum evolution (from the bottom to the top), as acquired by the interrogator, sampled each 3.4 s; (**b**) The estimated pressure is then reported as a function of elapsed time, with laser turned on at 0 s and turned off at 74 s.

The results of the first experiment are reported in Figure 4. The first chart shows the evolution of the EFPI spectrum with time, showing the optical spectrum as detected by the spectrometer, each 3.4 s. We observe, during the initial part of the acquisition, a blue shift of the EFPI envelope, which progressively saturates until the procedure is finished. On the right chart, the estimated pressure is reported. As the laser is turned on, pressure rapidly grows for the first 20 s, approaching 60 kPa. In the subsequent 40 s, pressure slowly increases, almost saturating to 70 kPa. The total ablation time is 74 s, with a peak pressure of 71.6 kPa; after that, laser is turned off and pressure rapidly decreases. In this experiment, where we position the pressure probe as close as possible to the LA applicator (0.5 mm), we observe a nearly instantaneous rise of pressure in correspondence of the ablation start; pressure then stabilizes to a nearly steady value.

**Figure 5:**
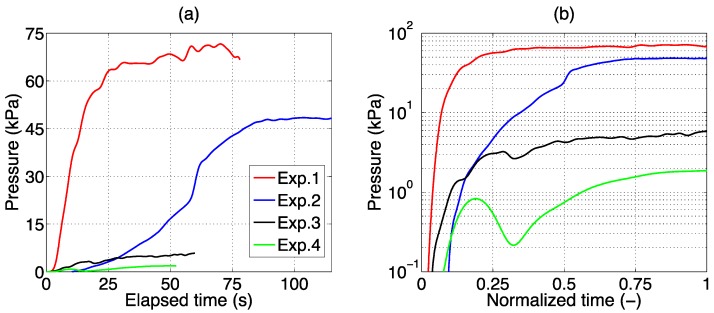
Evolution of pressure recorded by the EFPI probe, in four different LA experiments as outlined in Table 1. (**a**) Pressure chart for each experiment, reported from the start of the procedure to the final stage; (**b**) Pressure is reported, in logarithmic units, as a function of time, normalized between the values of 0 (laser on) and 1 (laser off).

Figure 5a shows the results of all four experiments, where pressure estimated by the EFPI is reported as a function of time. In the second experiment, having duration 41% longer and input power 40% inferior to the first one, we observe a pressure curve different from the previous experiment. In this case, pressure slowly increases for the first 58 s, up to 22 kPa; then, an abrupt rise is observed, reaching a steady-state value of 48 kPa after 80 s. The peak value is 48.4 kPa. In the last two experiments, as the tip-to-tip distance increases, pressure values are decreasing, as suggested in [25]: in the third experiment, pressure reaches a peak of 5.8 kPa, while in the last experiment, pressure changes appear to be minute (1.9 kPa peak).

As peak values are different and highly dependent upon the distance from the ablation tip, and ablation durations are different, Figure 5b proposes a comparison of all experiments, reporting the pressure in logarithmic units, and the time normalized between 0 (LA starts) and 1 (LA turned off). We observe the qualitative evolution of pressure throughout the experiments. In the first ablation, pressure rises quickly to reach a steady state approximately after 30% of the elapsed time. In the second experiment, a steady state is reached after more than half of the elapsed time. In the third experiment, with tip-to-tip distance of 2.5 mm, pressure has a steadier rise, and appears to settle to a steady-state value only at the end of the procedure. Finally, in the fourth experiment, in which the probe is perpendicular to the applicator, pressure exhibits a non-monotonic trend: after an initial rise, and a subsequent decrease, pressure rises slowly even after the laser source is turned off.

## 4. Discussion

**Figure 6:**
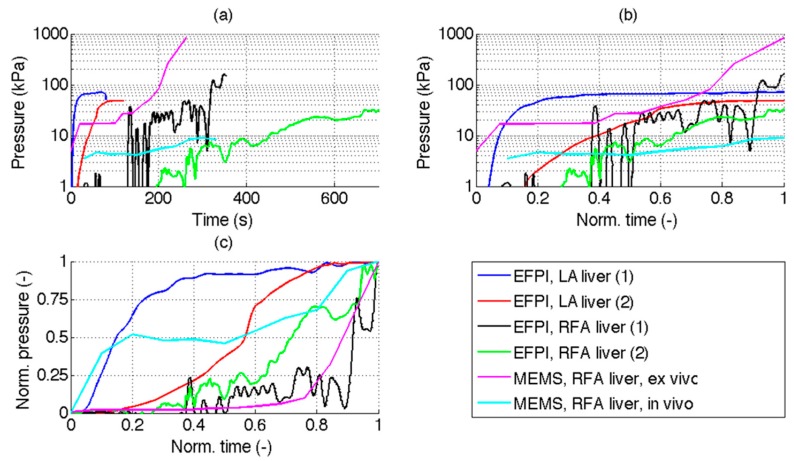
Comparison between experimental results and previous pressure measurements in mini-invasive TA, documented in literature. The chart compares EFPI pressure measurement performed in LA on hepatic tissue, reporting experiments 1 and 2 from Figure 5; experimental results obtained with a similar EFPI probe by Tosi *et al*. [25] on RFA ablation of liver, at 0.1 cm (1) and 0.5 cm (2) distance between the probe and the applicator; experimental *ex-vivo* results of Kotoh *et al*. [16], obtained with a MEMS sensor at 3 cm distance between the ablation device and probe; *in-vivo* study by Kotoh *et al*. [17] for hepatic RFA on animals, recorded with a MEMS sensor. Data are reported in three different formats: (**a**) Pressure (logarithmic units) as a function of time; (**b**) Pressure (logarithmic units) and normalized time; (**c**) Both pressure and time are normalized. Normalized data are within [0, 1] range whereas 0 corresponds to ablation start and 1 corresponds to peak pressure, and its corresponding elapsed time. MEMS experiments have been digitized from [16] and [17].

The present work provides, to the best of our knowledge, the first measurement of intra-tissue pressure in hepatic phantom for LA. We compare this result with the current literature related to pressure measurement in mini-invasive thermal ablation. Table 2 and Figure 6 compare the main results of our investigation, with the previous experimental results obtained by Tosi *et al*., with a similar EFPI probe, in RFA [25], and with results obtained for RFA by Kotoh *et al. ex vivo* [16] and *in vivo* [17]. Data from [21] is not included in the table and chart, due to the lack of information on the pressure sensor and temporal pressure charts. These, to the best of our knowledge, are the only results related to intra-tissue pressure measurement in mini-invasive thermal ablation designed for cancer care; all of them are related to hepatic tissue RFA.

In Table 1, the comparison among results achieved by current and previous studies is performed considering the peak-pressure value (*i.e.*, the maximum value of pressure measured at the end of the ablation procedure), and the 75% of peak (*i.e.*, normalized time for which the pressure value is 75% of the peak pressure). Moreover, the comparisons of pressure/time charts are reported in Figure 6, including: (a) original data; (b) time-normalized data; (c) normalization of both pressure and time.

In first place, we observe a disagreement in terms of methodology. In [15,16] the MEMS sensor does not withstand the four criteria for pressure sensor in TA, as in Section 1: the sensor has cylindrical shape having 1 cm diameter and 3 mm thickness, and the thermal error is estimated as 0.5 kPa/°C. In addition, in [15] the phantom in enclosed in a sealed capsule that contributes to artificially increase the detected pressure: as a consequence, the peak pressure reported in [15] is 833 kPa, while the same sensor records 9 kPa pressure *in vivo* [16]. The methodology proposed in the present study and in [23] is more suitable, as the probe has 0.2 mm thickness, yielding a negligible alteration of the ablation pattern [14], and the estimated thermal detuning is 0.03 kPa/°C: the low thermal error allows measuring the pressure at the point of treatment, in close proximity to the ablation peak, while [15,16] measured pressure in far field. The measurement is also more confined to a small volume, as the probe has 0.002 mm^2^ active volume, compared to 235 mm^2^ in [15,16]. On the other side, the EFPI probe is vulnerable to variations to refractive index outside of the active cavity, which often occurs during the pressure decrease after the TA procedure is discontinued.

The obtained results are in agreement with [25], and in partial disagreement with [16,17,21] due to the different methodology. In LA we observe a peak pressure of 71 kPa at 0.5 mm distance (tip-to-tip), and 48 kPa at 1.2 mm; in RFA we observe a peak pressure of 162 kPa at 1 mm distance, and 32 kPa at 5 mm distance. It is possible to conclude that pressure has a steep spatial gradient, which follows the steep thermal gradients often in excess of 3 °C/mm [2,8,14,15]. Hence, such differences between maximum pressure values during LA and RFA may be related to the different ablation pattern that the two sources induce: in fact, LA performed with one bare fiber is used to produce focal thermal lesion (maximum diameter of about 2 cm), whereas RFA allows obtaining larger ablation volumes (3 cm to 5 cm of diameter). These results appear in disagreement with [16], in which the pressure is recorded at 3 cm distance and 833 kPa peak is observed, but in reasonable agreement with [17] which records a peak value of 9 kPa at 15 mm distance. On the other side, recent studies on heat propagation in TA [26] appear to agree that the stress induced in the tissue is higher near the ablation tip(s), where heat is maximum.

An important factor for our analysis is the determination of the peak pressure value. To account for this parameter, which is shown in Figure 6c, we report in Table 2 the normalized time for which the 75% of the peak pressure is obtained. For LA, pressure reaches a steady-state relatively early, for normalized time of 0.24 at 0.5 mm tip-to-distance, and 0.63 at 1.2 mm distance; in RFA, instead, pressure is observed as a steep rise at the final ablation stage, when peak temperature approaches 100 °C, when the elapsed time is 83%–94% of the total ablation time.

As a challenge of mini-invasive TA is preventing scattered recurrences [18,19], the possibility of predicting the maximum intra-tissue pressure is therefore an asset. In RFA, highlighted as well in Figure 6b, a high peak pressure is observed; this is typically observed as a steep pressure rise in proximity of the water-to-vapor transition and such behavior makes it very hard to predict the final pressure value. Conversely, in LA our investigation shows that pressure reaches a steady value relatively early during the procedure; the closer the pressure probe is to the LA applicator, the earlier pressure reaches a regime condition. The possibility to better control pressure may give an additional advantage for LA, over other more established methods such as RFA, in the treatment of tumors. Furthermore, our investigation shows that in order to perform a predictive task, the probe has to be positioned as close as possible to the ablation tip, strengthening even more the requirements for minimum footprint and thermal insensitiveness.

This study sets the basis for further investigation of the parenchymal pressure measurement in LA, and its influence on the long-term lifetime expectance due to better control of scattered recurrences. A possible extension of this study in the field of LA is the investigation of pressure values during short-pulse laser-induced explosion of gold nano-particles injected within tumor, which allow selective nanophotothermolysis. The cellular damage is entailed by both temperature and vapour pressure produced by both water and gold vapours, therefore a fine control of pressure values can allow good performances of the treatment [6]. Moreover, this analysis can be also extended to other applications of laser ablation. For instance, lasers can be considered a valid alternative to scalpel in many different situations where it is crucial to limit collateral damage caused by electro-cautery [27]. In this scenario the abrupt heating of the tissue entails the generation and propagation of thermoelastic stresses. These pressure waves must be confined close to the cut (stress confinement), to optimize the healing process and cutting precision. Therefore, an analysis of tissue pressure may be useful to figure out the best laser settings (laser power, power, duration of pulse, and wavelength) to optimize the cutting.

## 5. Conclusions

In this work, we performed for the first time, to the best of our knowledge, a pressure measurement in laser ablation of hepatic tissue. Pressure is recorded with a miniature, biocompatible, and thermally insensitive fiber-optic probe based on EFPI. Experimental results have been obtained with different ablation parameters, and probe-applicator distance ranging from 0.5 mm to 5 mm. Measurements show peak values of 71.6, 48.4, 5.8, and 1.9 kPa at 0.5, 1.2, 2.5, and 5 mm tip-to-tip distance. In addition, pressure reaches a steady-state condition earlier, at shorter distances from the ablation device. A comparison with previous literature, based on fiber-optic sensors and MEMS for hepatic RFA, has been included. This investigation shows the premises for intra-tissue pressure measurement in LA, for improving tumor ablation by means of reduction of scattered recurrences.

## Figures and Tables

**Table 1:** Layout of LA experiments.

Experiment	Power (W)	Duration (s)	Tip-to-Tip Distance (mm)	Orientation	Peak Pressure (kPa)
1	5	74	0.5	Parallel	71.6
2	3	105	1.2	Parallel	48.4
3	4	60	2.5	Parallel	5.8
4	5	52	5	Perpendicular	1.9

**Table 2:** Comparison of intra-tissue pressure measurement methodologies and findings in mini-invasive thermal ablation for cancer care.

	Pressure Sensor		Thermal Ablation		Measurement			Results	
Study	Technology	Size	TA Type	Tissue	Distance Tip-to-Tip	Thermal Error ^1^	Peak Pressure ^2^		75% of Peak ^3^
[16]	MEMS	diameter 1 cm thickness 3 mm	RFA	Liver, porcine phantom	30 mm	0.5 kPa/°C	833 kPa		0.94
[17]	MEMS	diameter 1 cm thickness 3 mm	RFA	Liver, *in vivo* on pigs	15 mm	0.5 kPa/°C	9 kPa		0.83
[25]	Fiber optic EFPI	diameter 0.2 mm	RFA	Liver, porcine phantom	1 mm; 5 mm	0.03 kPa/°C	162 kPa; 32 kPa		0.93; 0.94
Current study	Fiber optic EFPI	diameter 0.2 mm	LA	Liver, porcine phantom	0.5 mm; 1.2 mm	0.03 kPa/°C	71 kPa; 48 kPa		0.24; 0.63

^1^ Data from [15,16] and sensor datasheet for MEMS; in-lab characterization for EFPI; ^2^ Values are reported for single-step ablation; ^3^ Normalized time for which the pressure value is 75% of the peak pressure.

## Abbreviations

The following abbreviations are used in this manuscript:

LA: Laser Ablation
TA: Thermal Ablation
RFA: Radiofrequency Ablation
MWA: Microwave Ablation
HIFU: High-Intensity Focused Ultrasound
OFS: Optical Fiber Sensors
MEMS: Micro Electro-Mechanical Systems
EFPI: Extrinsic Fabry-Perot Interferometer (EFPI)
SLED: Superluminescent LED

## References

[1] Goldberg S.N., Gazelle G.S., Mueller P.R. (2000). Thermal ablation therapy for focal malignancy: A unified approach to underlying principles, techniques, and diagnostic imaging guidance. Am. J. Roentgen..

[2] Dodd M.J. (2001). Radiofrequency ablation of the liver: current status. Am. J. Roentgen..

[3] Solbiati L., Livraghi T., Goldberg S.N., Ierace T., Meloni F., Dellanoce M., Cova L., Halpern E.F., Gazelle G.S. (2001). Percutaneous radio-frequency ablation of hepatic metastases from colorectal cancer: Long-term results in 117 patients. Radiology.

[4] Xu H.-X., Xie X.-Y., Lu M.-D., Chen J.-W., Yin X.-Y., Xu Z.-F., Liu G.-J. (2004). Ultrasound-guided percutaneous thermal ablation of hepatocellular carcinoma using microwave and radiofrequency ablation. Clin. Radiol..

[5] Kennedy J.E. (2005). High-intensity focused ultrasound in the treatment of solid tumours. Nat. Rev. Cancer.

[6] Letfullin R.R., Joenathan C., George T.F., Zharov V.P. (2006). Laser-induced explosion of gold nanoparticles: Potential role for nanophotothermolysis of cancer. Nanomedicine.

[7] Di Costanzo G.G., Francica G., Pacella C.M. (2014). Laser ablation for small hepatocellular carcinoma: State of the art and future perspectives. World J. Hepatol..

[8] Saccomandi P., Schena E., Caponero M.A., Di Matteo F.M., Martino M., Pandolfi M., Silvestri S. (2012). Theoretical analysis and experimental evaluation of laser-induced interstitial thermotherapy in *ex vivo* porcine pancreas. IEEE Trans. Biomed. Eng..

[9] Papini E., Guglielmi R., Bizzarri G., Graziano F., Bianchini A., Brufani C., Pacella S., Valle D., Pacella C.M. (2007). Treatment of benign cold thyroid nodules: A randomized clinical trial of percutaneous laser ablation versus levothyroxine therapy or follow-up. Thyroid.

[10] Schroeder J.L., Missios S., Barnett G.H, Mohammadi A.M. (2014). Laser interstitial thermal therapy as a novel treatment modality for brain tumors in the thalamus and basal ganglia. Photonics Las. Med..

[11] Di Matteo F.M., Picconi F., Martino M., Pandolfi M., Pacella C.M., Schena E., Costamagna G. (2014). Endoscopic ultrasound-guided Nd:YAG laser ablation of recurrent pancreatic neuroendocrine tumor: A promising revolution?. Endoscopy.

[12] Lee S.H., Riu Y.T., Son D.H., Jeong S., Kim Y., Ju S., Kim B.H., Han W.T. (2015). Radial-firing optical fiber tip containing conical-shaped air-pocket for biomedical applications. Opt. Express.

[13] BCC Research Ablation Devices: Technologies and Global Markets—HLC163A. http://www.bccresearch.com/.

[14] Saccomandi P., Schena E., Silvestri S. (2013). Techniques for temperature monitoring during laser-induced thermotherapy: An overview. Int. J. Hypertherm..

[15] Tosi D., Macchi E.G., Gallati M., Braschi G., Cigada A., Rossi S., Leen G., Lewis E. (2014). Fiber-optic chirped FBG for distributed thermal monitoring of *ex-vivo* radiofrequency ablation of liver. Biomed. Opt. Express.

[16] Kotoh K., Nakamuta M., Morizono S., Kohjima M., Arimura E., Fukushima M., Enjoji M., Sakai H., Nawata H. (2005). A multi-step, incremental expansion method for radio frequency ablation: optimization of the procedure to prevent increases in intra-tumor pressure and to reduce the ablation time. Liver Int..

[17] Kotoh K., Morizono S., Kohjima M., Enjoji M., Sakai H., Nakamuta M. (2005). Evaluation of liver parenchymal pressure and portal endothelium damage during radio frequency ablation in an in vivo porcine model. Liver Int..

[18] Mulier S., Ruers T., Jamart J., Michel G., Marchal G., Ni Y. (2008). Radiofrequency ablation versus resection for resectable colorectal liver metastases: Time for a randomized trial?. Dig. Surg..

[19] Inokuchi R., Seki T., Ikeda K., Kawamura R., Asayama T., Yanagawa M., Umehara H., Okazaki K. (2010). Percutaneous microwave coagulation therapy for hepatocellular carcinoma: Increased coagulation diameter using a new electrode and microwave generator. Oncol. Rep..

[20] Kotoh K., Arimura E., Morizono S., Kohjima M., Enjoji M., Sakai H., Nakamuta M. (2005). Scattered and rapid intrahepatic recurrences after radio frequency ablation for hepatocellular carcinoma. World J. Gastroenterol..

[21] Kim H.J., Rhim H., Lee M.W., Jeong W.K. (2015). Measurement of intrahepatic pressure during microwave ablation in an *ex vivo* bovine liver model. Gut Liver.

[22] Roriz P., Frazao O., Lobo-Ribeiro A.B., Santos J.L., Simoes J.A. (2013). Review of fiber-optic pressure sensors for biomedical and biomechanical applications. J. Biomed. Opt..

[23] Bae H., Yu M. (2012). Miniature Fabry-Perot pressure sensor created by using UV-molding process with an optical fiber based mold. Opt. Express.

[24] Nakamuta M., Kohjima M., Morizono S., Yoshimoto T., Miyagi Y., Sakai H., Enjoji M., Kotoh K. (2006). Comparison of tissue pressure and ablation time between the LeVeen and cool-tip needle methods. Comparat. Hepatol..

[25] Tosi D., Macchi E.G., Braschi G., Cigada A., Gallati M., Rossi S., Poeggel S., Leen G., Lewis E. (2014). Fiber optic combined FPI/FBG sensors for monitoring of radiofrequency thermal ablation of liver tumors: *Ex-vivo* experiments. Appl. Opt..

[26] Chu K.F., Dupuy D.E. (2014). Thermal ablation of tumours: Biological mechanisms and advances in therapy. Nat. Rev. Cancer.

[27] Franjic K., Cowan M.L., Kraemer D., Miller R.J. (2009). Laser selective cutting of biological tissues by impulsive heat deposition through ultrafast vibrational excitations. Opt. Express.

